# Diagnostic parallels between borderline and bipolar patients in psychopathology: Similarities, differences, comorbidities, neural correlates, and a new proposal for the Perrotta Border‐Bipolar Profile Diagnostic Questionnaire

**DOI:** 10.1002/ibra.70001

**Published:** 2025-09-02

**Authors:** Giulio Perrotta, Simona Grilli, Stefano Eleuteri, Irene Petruccelli

**Affiliations:** ^1^ Department of Psychology Universitas Mercatorum Rome Italy

**Keywords:** bipolar spectrum disorder, borderline personality disorder, depression, mania, PICI‐3

## Abstract

In psychopathology, one of the most complex challenges is offered by the diagnostic comparison between patients with borderline personality disorder and those with bipolar spectrum disorder, as some of the symptoms characterizing the two disorders overlap. This detail can mislead therapists who are often called upon to diagnose these syndromes, and in some cases, there is a tendency to diagnose both as one is a personality disorder and the other is a mood disorder. This article provides the reader with a complete overview of these two disorders, highlighting some aspects that could redefine their clinical framework. This need could favor a reduction in cases of diagnostic error, also through the proposal of an innovative psychometric tool, Perrotta Border‐Bipolar Profile Diagnostic Questionnaire, currently being validated.

## Graphical abstract

In psychopathology, one of the most complex challenges is offered by the diagnostic comparison between patients with borderline personality disorder and those with bipolar spectrum disorder, as some of the symptoms characterizing the two disorders overlap. Neurobiological studies on these disorders give pause for thought that the current clinical interpretive approach may not be effective and therefore needs reevaluation. This paper provides the reader with a comprehensive overview of these disorders, highlighting some aspects that could redefine their clinical framing, thus facilitating a better diagnostic approach to the problem and offering an innovative key that could eliminate the problem of diagnostic error at the root, through the proposal of a questionnaire (Perrotta Border‐Bipolar Profile Diagnostic Questionnaire), which is in the validation phase, that could refine the diagnosis in the hypotheses of borderline disorder and bipolar spectrum disorder.

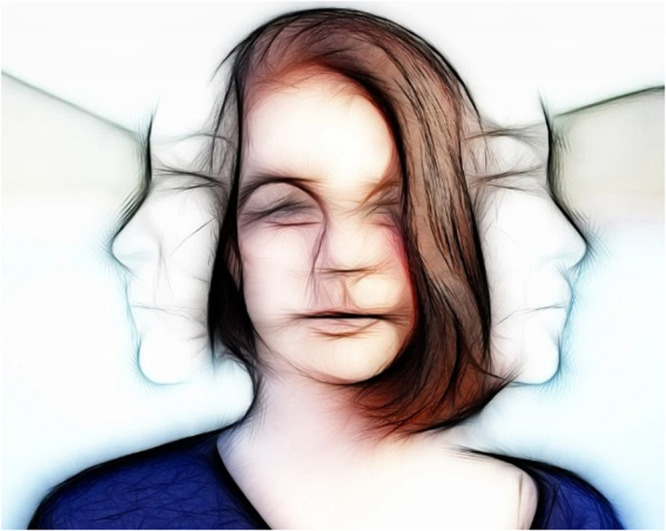

## 1 INTRODUCTION

In psychopathology, on both the psychology and psychiatry fronts, one of the most complex and inspiring challenges is offered by the diagnostic parallelism between patients with borderline personality disorders (BPD) and those with bipolar spectrum disorders (BSD), as many of the symptoms seem to overlap, leading to confusion. This detail can mislead therapists who are often called upon to diagnose these syndromes, and some of them even see the possibility of a combined diagnosis, by the similarities and assumptions of comorbidity. Such assessment, however, is often subject to subjective interpretive judgment based on clinical history, narrative during interviews, and psychodiagnostic outcomes, which to date have never truly defined the boundary between the diagnosis of BPD and that of BSD, although the two nosographic categories are distinct. This opacity is brought about by the fact that some symptoms described by patients are identical, and therefore, it is very difficult to draw a clear line of demarcation. It is not uncommon, then, to witness combined diagnoses, labeling the patient both bipolar and borderline, without a clear diagnostic structure, also because of the best psychotherapeutic and psychopharmacological profile to be applied.^1^, ^2^, ^3^, ^4^

The reasons for these critical issues may be justified by their nosographic nature,^5^ as the symptoms described in each of the two disorders have peculiar similarities, such as the marked impulsivity, acceleration, and aggression of the borderline and bipolar in the manic phase. However, in the current scientific setting, the two disorders are kept separate because borderline is a personality disorder, while bipolar is a mood disorder, and therefore, they may also overlap and be diagnosed together. This approach, while simplistic, is refuted in clinical practice, as demonstrated in the Perrotta Integrative Clinical Interviews – 3 (PICI‐3) model,^6^ which instead considers both as personality disorders and therefore overlap is possible but only partially, based on dysfunctional personality traits.

The need, therefore, to propose this new interpretive framework stems from the fact that the partial symptomatological overlap in the world literature underestimates the risk of diagnostic error, as the psychotherapy of the borderline is not that of the bipolar, as traced in clinical manuals; moreover, the psychopharmacological approach also changes and therefore highlighting the diagnosis in both a structural and functional framework, with reference to personality traits, typical of PICI‐3, appears more coherent, structured, safe and precise than the current interpretative system that in several cases posits an egalitarian diagnosis of BSD and BPD.

The present work aims to provide the reader with a comprehensive overview of these disorders, highlighting some aspects that could redefine their clinical framing, thus facilitating a better diagnostic approach to the problem and offering an innovative key that could eliminate the problem of diagnostic error at the root, through the proposal of a questionnaire to be validated that could refine the diagnosis in the hypotheses of BPD and BSD. Therefore, the primary purpose of this paper is to provide a structured and functional overview of the diagnostic phenomenon and the similarities and differences between the two nosographic categories, while the secondary purpose is to provide mental health professionals with a psychometric tool, to be validated, that would definitively clarify the guiding framework and interpretive rules in the case of patients with these disorders.

## 2 SEARCH STRATEGY

**Figure 1:**
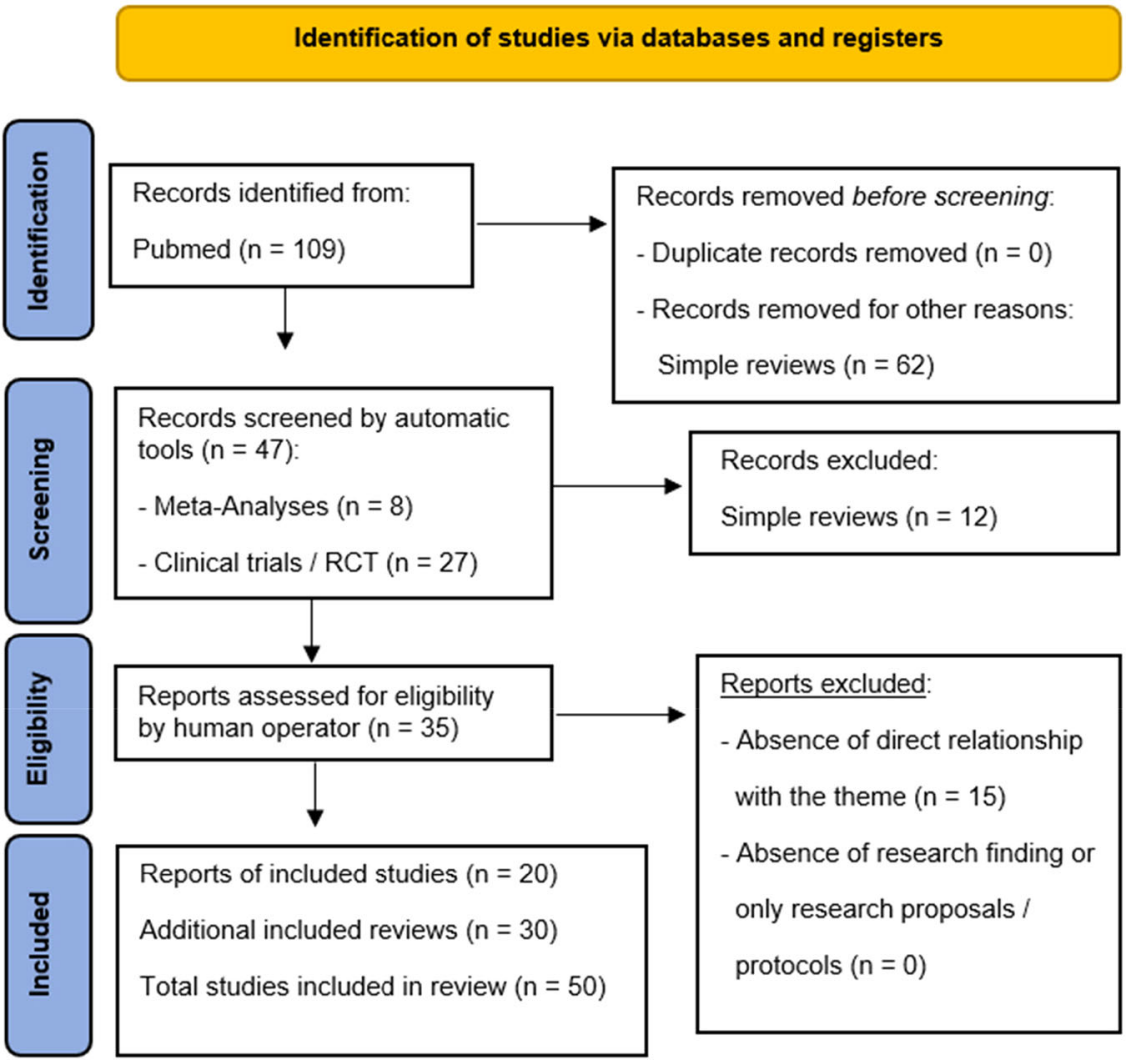
The Preferred Reporting Items for Systematic Reviews and Meta‐Analyses (PRISMA) flow diagram for this systematic review. [Color figure can be viewed at wileyonlinelibrary.com]

The author searched PubMed, from January 2014 to March 2024, for meta‐analyses, clinical trials and randomized controlled trials using the binomial “Borderline AND Bipolar”, selecting 109 eligibility results, to collect the updates of the last decade. To have a greater and complete overview of the topic, we ultimately selected a total of 20 studies, still adding 30 more reviews to be able to argue the elaborated content (to more easily contextualize definitions and clinical‐diagnostic profiles), for an overall total of 50 results. Simple reviews, opinion contributions, or publications in popular volumes were excluded because they were not relevant or redundant for this study. The search was limited to Italian and English‐language papers (Figure 1).

## 3 THE NOSOGRAPHIC PROFILE ACCORDING TO THE DIAGNOSTIC AND STATISTICAL MANUAL OF MENTAL DISORDERS, FIFTH EDITION, TEXT REVISION (DSM‐5‐TR)

### 3.1 General aspects

The DSM‐5‐TR is the most comprehensive and up‐to‐date tool for clinical practice available to practitioners and researchers in the field of mental health; with contributions from more than 200 experts, this source offers the most current findings in the scientific literature and provides an organic presentation of diagnostic criteria, coding systems, and descriptions of disorders. From prevalence statistics to associated characteristics, from development and course to risk factors and prognosis, and diagnostic markers: the entire text has been recently revised, updated, and innovated, demonstrating particular attention to sociocultural framing, gender issues, and issues related to suicidal ideation and behavior, topics addressed crosswise throughout the volume.^7^

### 3.2 BPD according to the DSM‐5TR

The DSM‐5‐TR defines BPD as one of the 4 cluster B personality disorders characterized by a consistent, pervasive, and enduring pattern of instability in interpersonal relationships, self‐image, and affectivity, and marked impulsivity. Onset generally occurs by late adolescence and early adulthood, when the disorder manifests in a variety of socio‐familial contexts. Variability and heterogeneity are pathognomonic features of BPD: variability means that no trait is always present and that periods of distress oscillate with phases of well‐being and good social adjustment, in a clinical picture that can change rapidly due to hyperactivating events; heterogeneity means the need to meet at least five out of nine criteria for diagnosis. BPD is characterized by the dysfunctionality of at least two of the four domains of an individual's psychosocial functioning: cognitive, emotional/affective, relational, and behavioral (particularly reflex or impulsive behavior); as far as the cognitive domain is concerned, then, two subdomains are mainly impaired: the belief system regarding self‐image and the ability to regulate one's emotions.^8^ As already stated, to make a diagnosis of BPD, it is necessary for the patient to consistently manifest at least five of the following nine symptoms^9^, ^10^, ^11^:

(a) Desperate efforts (excluding threats, suicide attempts, and self‐harming acts) to avoid abandonment, real or imagined, by a significant person. They experience intense fears of abandonment and inappropriate anger even when facing real‐time‐limited separations or when unavoidable changes in plans intervene (e.g., sudden despair as a reaction to the therapist's announcement that interview time is over; panic or fury when someone important to them is a few minutes late or must cancel an appointment). They may believe that this “abandonment” implies that they are “bad.” These fears of abandonment are associated with the intolerance of being alone and the need to have people with them.
(b) A constant pattern of interpersonal relationship instability. They may idealize reference figures or potential lovers at the first or second meeting, ask to spend a lot of time together, and share intimate details at the beginning of a relationship. However, they may move quickly from idealizing other people to devaluing them, feeling that the other person does not care enough about them, does not give enough, or is not “present” enough.
(c) Identity alteration: marked and constant instability of self‐image and self‐concept. It is equivalent to poor self‐knowledge. The borderline subject is unable to fully and coherently describe to himself or others the characteristics of his personality and character. There may be sudden changes in opinions and plans about career, sexual identity, values, and types of friends. They may suddenly shift from the role of victim, in need of help, to that of avenger of previous mistreatment. Although they usually have a self‐image based on being bad or dangerous, they may sometimes feel that they do not exist at all.
(d) Impulsivity in at least two types of behavior (examples of behavior: reckless spending, promiscuous and/or compulsive sex, substance and/or behavioral abuse, reckless driving, binge drinking).
(e) Recurrent threats and/or attempts to commit suicide and self‐injurious acts. Suicide occurs in 8%–10% of cases, and self‐injurious acts (e.g., cutting or burning oneself) and suicide threats and attempts are very common. Suicidal tendency is often the reason for seeking therapeutic help.
(f) Instability of affectivity due to marked mood reactivity (e.g., dysphoria, irritability, or anxiety, of short duration from a few hours to no more than two/three days). It can be confusing with some mood disorders (BSD or cyclothymia), but in BPD, the transition between depression and mania/hypomania is much more rapid and fleeting.
(g) Chronic feeling of emptiness, of feeling insignificant. Easily bored, borderline individuals may constantly be looking for something to do.
(h) Inappropriate anger concerning the context, both manifested and intentional, intense and constant, with frequent extremes of anger and both verbal and physical confrontations.
(i) Paranoid episodes of short duration, mainly due to stressors, as well as dissociative episodes, even of severe intensity, but these are generally of insufficient severity or duration to warrant an additional diagnosis. These episodes occur most frequently in response to real or imagined abandonment.
  Based on the prevalent traits, four types of borderlines have been proposed.^6^, ^9^ These are theoretical models in which, of course, there may be overlaps between the various borderline personality types:

(a) Borderline dependent personality disorder: characterized by clingy and dependent behavior, individuals in this group are overly accommodating to the needs of others and have difficulty setting boundaries. They seek approval, but also tend to avoid people, feel unworthy, and may tend toward depression.
(b) Impulsive BPD: although lack of impulse control is a typical symptom of borderline disorder, in the case of impulsive BPD, this trait is further exaggerated. The impulsive type involves a loss of control not in emotions, but in behaviors. This increases the risk of substance abuse, self‐injurious behaviors, substance abuse, and risky sex.
(c) Borderline aggressive personality disorder: “inappropriate,” or “uncontrollable” anger, unpredictability, irritability, defiance, and impatience characterize this type of borderline disorder. They tend to be passive‐aggressive in relationships and may exhibit self‐injurious behaviors to attract attention. Often this manifestation of anger leads to serious relationship problems, complaints, and problems maintaining job positions.
(d) Self‐destructive BPD: they are bitter. They have no sense of self and are terrified of being abandoned. They may hurt themselves by proving something. They are prone to risky behaviors such as reckless driving and promiscuous sexual activity.

BPD has comorbidity with anxiety disorders, obsessive‐compulsive disorders, eating disorders, substance use disorder and other behavioral disorders (addictions), impulse control disorders, sleep disorders, mood and psychotic states, and other personality disorders (especially of the same cluster B).^9^, ^12^

### 3.3 BSD according to the DSM‐5TR

The DSM‐5‐TR defines BSD as a spectrum of mood disorders that originated from the generic term “manic‐depressive psychosis” and consisted of syndromes of psychiatric interest characterized by an alternation between the two counter‐polar conditions of psychic activity, its excitement (mania) and, on the reverse, its inhibition (depression), combined with a wide range of neurotic symptoms and psychotic alterations in thinking. It is therefore not a personality disorder, as is the case with borderline disorder. Functional dysregulation results in the development of alterations in thymic balance (mood disorders), ideational processes (alterations in the form and content of thought), motor and behavioral initiative, and neurovegetative manifestations (abnormalities in energy levels, appetite, sexual desire, sleep‐wake cycle‐rhythm).^13^

**Figure 2:**
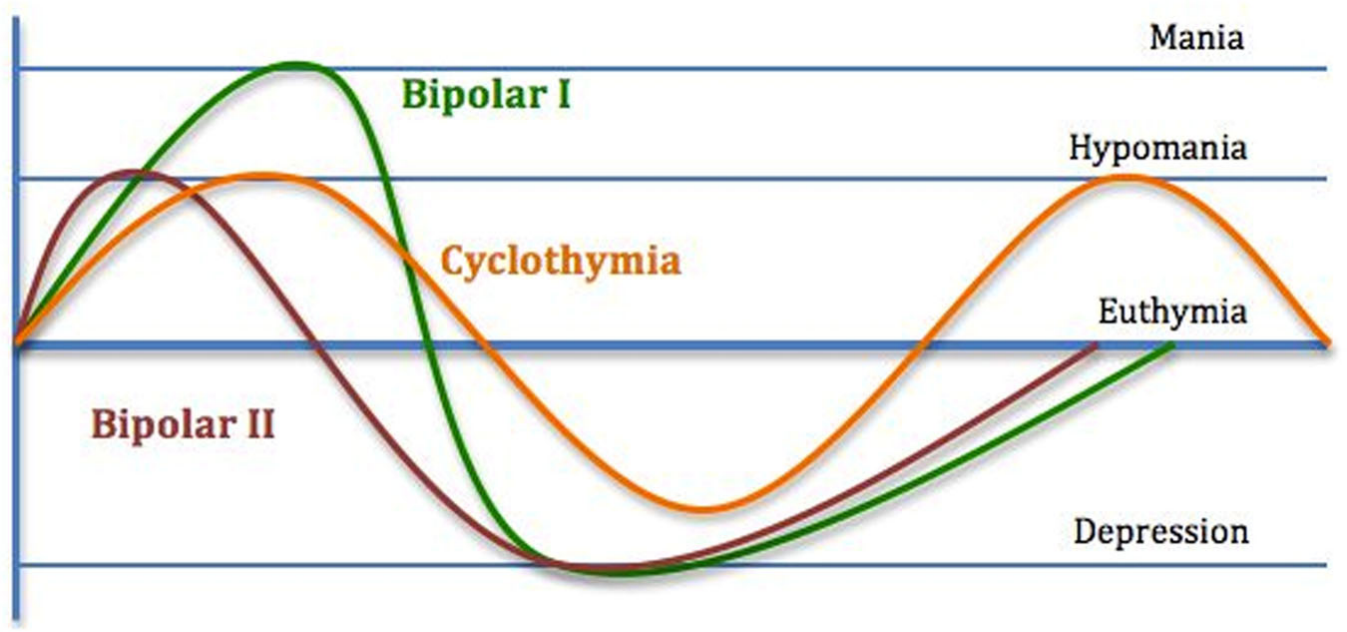
Polarization curve in bipolar spectrum disorders, according to the DSM‐5‐TR. This figure describes all the possible mood curves of depressive disorder, from normal to pathological functioning. [Color figure can be viewed at wileyonlinelibrary.com]

The complexity of the bipolar diagnosis is determined by the fact that it has alternating episodes of mania and depression in its pathological structure, although many patients have a predominance of one or the other. BSD are classified as,^14^, ^15^, ^16^, ^17^, ^18^, ^19^, ^20^ Figure 2]:

(a) Type I BSD: it is defined by the presence of at least one full‐blown manic episode (i.e., causing marked impairment in social or occupational function or including delusions) and usually depressive episodes (in fact, a full diagnosis of depressive disorder is not necessary for the purpose of the diagnosis of BSD).
(b) Type II BSD: it is defined by the presence of episodes of major depression with at least one hypomanic episode, but no true manic episodes.
(c) Substance/drug‐induced BSD: it is defined by the presence of a mood disorder compatible with mania that develops during or immediately after exposure to, intoxication with, or weaning from a substance (e.g., cocaine, corticosteroids) capable of producing such symptoms.
(d) BSD and related disorders due to another medical condition: they are defined by the presence of a mood disorder compatible with mania caused by a pharmacological condition (e.g., Cushing's syndrome or head injury) and not occurring exclusively during an episode of delirium.
(e) Not otherwise specified BSD: it is characterized by overt bipolar features that do not meet the specific criteria for one of the other BSD in cyclothymic disorder, in which patients have prolonged (>2 years) periods that include both hypomanic and depressive episodes; however, these episodes do not meet the specific criteria for a major depressive disorder or BSD.

BSD begins with an acute phase of symptoms of depression or mania, followed by a repetition of remissions and relapses. Remissions are often complete, but many patients have residual symptoms, and for some, the ability to function at work is severely impaired. Relapses are distinct episodes of more intense symptoms that are manic, depressive, hypomanic, or a mixture of depressive and manic features. Episodes last from a few weeks to 3–6 months; generally, depressive episodes last longer than manic or hypomanic episodes. Cycles, the time between the onset of one episode and the onset of the next, vary concerning duration from patient to patient. Some patients have frequent episodes, perhaps only a few over a lifetime, while others have rapidly cycling forms (usually defined as ≥4 episodes/year). Only a minority alternate between mania and depression each cycle; in most cases, one or the other predominates. Patients may attempt or complete suicide. A manic episode is defined as ≥1 week of persistently elevated, euphoric or irritable mood and increased goal‐directed activity or a marked increase in energy plus ≥3 additional symptoms (≥4 if the mood is only irritable), according to the current DSM‐5‐TR description. Manic psychosis is a more extreme manifestation, with psychotic symptoms that can be difficult to distinguish from schizophrenia. Patients may have extreme delusions of grandeur or persecutory delusions, sometimes with hallucinations. A hypomanic episode, on the other hand, is a less extreme form of mania, which includes a distinct episode lasting ≥4 days with behavior that is markedly different from the patient's usual nondepressed self and includes ≥3 of the additional symptoms listed above in mania. During the hypomanic period, mood is elevated as energy increases markedly, the need for sleep decreases, and psychomotor activity increases. For some patients, hypomanic periods are adaptive because they give great energy, creativity, confidence, and higher‐than‐normal social functioning; however, for others, both hypomania and mania can be markedly maladaptive. A major depressive episode, on the other hand, has typical features of major depression; the episode must include ≥5 of the following symptoms in the same 2‐week period, and one of them must be depressed mood or loss of interest or pleasure and, except for suicidal thoughts or attempts, all symptoms must be present almost every day: depressed mood for most of the day, marked decrease in interest or pleasure in all or most activities for most of the day, significant (>5%) weight gain or loss or decrease or increase in appetite, insomnia (often maintenance insomnia) or hypersomnia, psychomotor agitation or slowing observed by others (not self‐reported), asthenia or loss of energy, excessive or inappropriate feelings of self‐evaluation or guilt, decreased ability to think or concentrate or indecision, recurrent thoughts of death or suicide, suicide attempt, or specific plans to enact it. Psychotic features are more common in bipolar depression than in unipolar depression. Finally, mixed features are the most complex to diagnose and have a worse prognosis than a pure manic or hypomanic state, with a significantly increased risk of suicide.^21^, ^22^, ^23^ According to the DSM‐5‐TR, a comparative analysis of BPD and BSD is presented in Table 1.

**Table 1:** Comparison of borderline personality disorder (BPD) and bipolar spectrum disorder (BSD), according to the Diagnostic and Statistical Manual of Mental Disorders, Fifth Edition, Text Revision (DSM‐5‐TR).^6^, ^9^

Variable	Borderline personality disorder (BPD)	Bipolar spectrum disorder (BSD)
*Cluster of disorder*	Personality disorder (Cluster B)	Mood spectrum disorder
*Types of disorder*	Type 1 (dependent)	Type 1 (manic‐depressive)
	Type 2 (impulsive)	Type 2 (mild form)
	Type 3 (aggressive‐explosive)	Type 3 (mixed)
	Type 4 (self‐destructive)	Type 4 (from substances or disease)
		Type 5 (not otherwise specified)
*Typology of symptoms*	The constant, pervasive and enduring pattern of instability in interpersonal relationships, self‐image and affectivity, and marked impulsiveness	The spectrum of humoral disorders consists of the alternation between the two counter‐polar conditions of psychic activity, its excitement (mania) and, on the revers,e its inhibition (depression), combined with a wide range of neurotic symptoms and psychotic alterations in thinking
*Frequency of symptoms*	Repeated and close‐up	Cyclic and of prolonged duration
*Dysfunctional symptoms*:		
1. Fear of abandonment	Present	Absent
2. Unstable relationships	Present	Present (only in the mania)
3. Unstable self‐image	Present	Present (only in the mania)
4. Affective lability	Present	Present
5. Impulsivity	Present	Present (only in the mania)
6. Use of aggressive or violent acts	Present	Present (only in the mania)
7. Irritability and instability humoral	Present	Present (only in the mania)
8. Idealization/devaluation	Present	Present (only in the mania)
9. Low empathy	Present	Present (only in the mania)
10. Chronic feelings of sadness, emptiness, delusion, and boredom	Present	Present (only in the depression)
11. Tendency toward drama and exaggeration	Present	Present (only in the mania)
12. Inappropriate and disproportionate anger	Present	Present (only in the mania)
13. Risky behaviors	Present	Present
14. Psychotic manifestations (paranoia and dissociation)	Present	Present
15. Manic episode/hypomanic	Present	Present (only in the mania)
16. Depressive episode/dysthymic	Present	Present (only in the depression)
17. Cyclothymia	Present	Present (only in the depression)

### 3.4 Bipolar‐borderline disorders co‐diagnosis (BB‐co) according to the DSM‐5‐TR

According to the DSM‐5‐TR nosographic formulation, these two disorders can coexist in the same patient, as a mood disorder (BSD) is grafted into the personality disorder (border). It happens that a borderline subject presents alongside the affective instability proper to the disorder, true depressive, or manic episodes. In such a case, we have a comorbidity between borderline disorder and BSD. In detail, it appears that about 20% of patients with borderline disorder are also affected by BSD, and 15% of patients with BSD are also affected by BPD. This high prevalence of borderline bipolar comorbidity further complicates the diagnosis. However, this clinical approach is refuted by the PICI model, as enunciated in the next section.^6^, ^9^, ^24^, ^25^, ^26^, ^27^, ^28^, ^29^

## 4 THE NOSOGRAPHIC PROFILE ACCORDING TO THE PICI‐3

### 4.1 General aspects

PICI‐3 was created and structured into four sections, dedicated to dysfunctional traits in children and pre‐adolescents (PICI‐C‐3, 8–13 years) and in adolescents, adults, and the elderly (PICI‐TA‐3, 14–90 years), to common secondary disorders (PICI‐DS‐3, 8–90 years) and functional traits (PICI‐FT‐3, 8–90 years), with the identification of all functional elements and structural aspects of personality according to the model underlying the PICI (IPM) which identifies mental disorders into personality disorders, dividing them into 25 different types. PICI‐3 is a valid, efficient, and effective psychometric tool to identify the functioning or dysfunction of personality traits for psychopathological diagnosis.^6^ Notably, compared to DSM‐5‐TR, due to the habitual, stable, persistent, and pervasive nature of mood fluctuations, the PICI‐3 framework classifies BSD as a personality disorder, referred to as bipolar personality disorder (BpD).

### 4.2 BPD according to the PICI‐3

The PICI‐3 defines BPD (category No. 11) as a habitual, stable, persistent, and pervasive pattern, with onset around age 8 (but evolving structurally into adolescence and adulthood), characterized by emotional instability, sudden mood swings, and impulsivity. The model under review identifies 9 specific dysfunctional traits of this disorder: 11.1) emotional instability and/or impulsivity in interpersonal relationships; 11.2) sudden mood swings; 11.3) active and/or passive manipulative tendency; 11.4) desperate efforts to avoid abandonment (real and/or imagined); 11. 5) dysfunctional and/or unstable self‐image; 11.6) marked impulsivity capable of harming them; 11.7) persistent feelings of emptiness; 11.8) sudden anger and unwarranted aggression; 11.9) irrational thoughts and beliefs, leading wholly or partly into the psychotic sphere. Again, this model seeks to frame the disorder according to a precise rating scale, starting from its characteristic traits, drawing a psychodiagnostic profile that tends from a simple characterization typical in highly functional elevations to the highest level of dysfunction and pathological impairment; in particular, the diagnosis will be based on the level of insight present and how many specific dysfunctional traits emerge in the patient's personological picture, according to the “Perrotta Borderline Disorder Severity Scale”.^9^ According to this scale: 1) excellent, if it has 5 traits; 2) good, if it has 6 traits; 3) mediocre, if it has 7 traits; 4) low, if it has 8 traits; 5) poor, if it has 9 traits. The model under review preferred not to subdivide the disorder into subcategories because it is structured based on the impact of individual dysfunctional traits on the patient's life and therefore the presence or absence of one or more traits automatically defines the specific type (e.g., if the traits above are more aggressive it will be defined as an aggressive borderline) among the following categories: unstable‐impulsive (traits 11. 1 and 11.5), aggressive‐explosive (traits 11.3, 11.6, and 11.8), emotional‐humoral (traits 11.2 and 11.4), deflected (traits 11.2 and 11.7), fragmented (traits 11.5 and 11.9), and mixed (when a heterogeneous combination of traits results).^6^

### 4.3 BpD according to the PICI‐3

**Figure 3:**
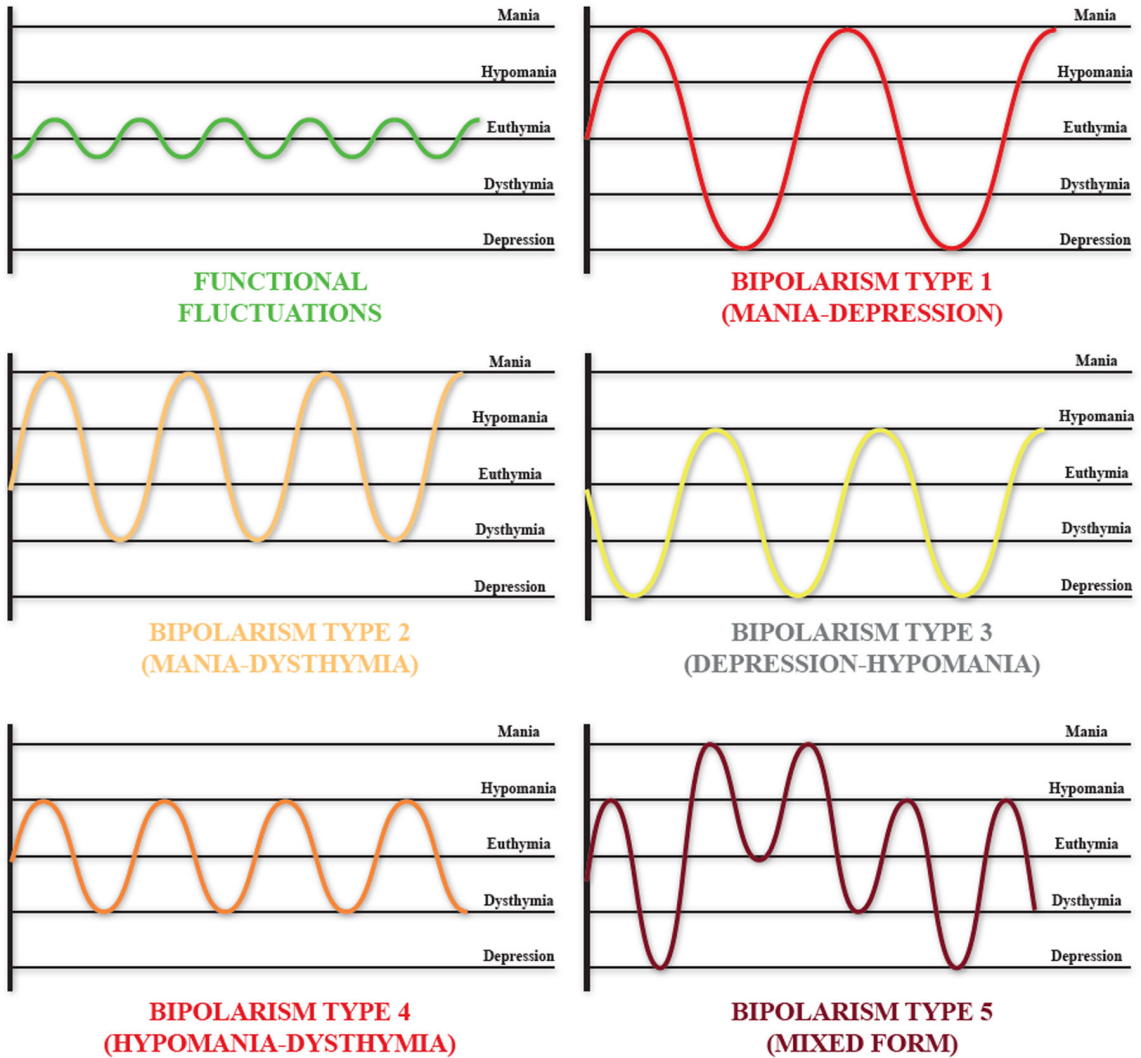
Polarization curve in bipolar personality disorder (BpD), according to PICI‐3. [Color figure can be viewed at wileyonlinelibrary.com]

PICI‐3 defines BpD (category No. 7) as a habitual, stable, persistent, and pervasive pattern, with onset between the ages of 5 and 10 years (but evolves structurally into adolescence), characterized by abrupt mood fluctuations, manic and/or depressive states, and/or abrupt alternation and emotional instability. The model under review identifies 9 specific dysfunctional traits of this disorder: 7.1) abrupt mood fluctuations; 7.2) emotional instability; 7.3) relational and/or social instability; 7.4) manic, depressive and/or mixed episodes; 7.5) tendency to active and/or passive manipulation; 7. 6) low tolerance to frustration and anxiety; 7.7) tendency to irritability; 7.8) low tolerance to criticism; 7.9) mood tends to be dysphoric (with or without unpleasant feelings, frustration, pessimism, tension, irritability, anxiety and psychomotor agitation). By the DSM‐5‐TR model, PICI‐3 also opted for structural classification of BpD, identifying based on dysfunctional traits, however, five main forms: a) Type I bipolarity: overt alternation of manic episodes and depressive episodes; b) Type II bipolarity: overt alternation of manic episodes and dysthymic episodes; c) Type III bipolarity: collimated alternation of hypomanic episodes and depressive episodes; d) Type IV (or cyclothymic or cyclothymia) bipolarity: collimated alternation of hypomanic episodes and dysthymic episodes; e) Type V (or mixed) bipolarity: mixed alternation that cannot be defined or differently framed (Figure 3).

Let's finally clarify the terminology: “bipolar spectrum disorders” or “bipolarity”? We use the term “bipolar spectrum disorders” when referring to the clinically relevant conditions (i.e., the specific disorder), while we use the term “Bipolarity” when referring to one of the two extreme poles, manic or depressive, to explain the patient's dysfunctional orientational set‐up.^6^ BPD and BpD are also compared based on PICI‐3 (Table 2).

**Table 2:** Comparison of borderline personality disorder (BPD) and bipolar personality disorder (BpD), according to the Perrotta Integrative Clinical Interviews ‐ 3 (PICI‐3).^6^, ^9^

Variable	Borderline personality disorder (BPD)	Bipolar personality disorder (BpD)
*Cluster of disorder*	Personality disorder (Cluster B)	Personality disorder (Cluster B)
*Types of disorder*	Type 1 (Unstable‐impulsive)	Type 1 (Mania‐Depression)
	Type 2 (Aggressive‐explosive)	Type 2 (Mania‐Dysthymia)
	Type 3 (Emotional‐humoral)	Type 3 (Depression‐Hypomania)
	Type 4 (Deflected)	Type 4 (Hypomania‐Dysthymia)
	Type 5 (Fragmented)	Type 5 (Mixed form)
	Type 6 (Mixed)	
*Typology of symptoms*	The constant, pervasive, and enduring pattern of instability in interpersonal relationships, self‐image, and affectivity, and marked impulsiveness	The spectrum of humoral disorders consists of the alternation between the two counter‐polar conditions of psychic activity, its excitement (mania) and, on the reverse, its inhibition (depression), combined with a wide range of neurotic symptoms and psychotic alterations in thinking
*Frequency of symptoms*	Repeated and close‐up	Cyclic and of prolonged duration
*Dysfunctional symptoms*:		
1. Emotional/social instability	Present	Present
2. Impulsiveness	Present	Present (only in the mania)
3. Sudden mood swings	Present	Present
4. Manipulative tendency	Present	Present
5. Desperate efforts to avoid abandonment	Present	Absent
6. Dysfunctional and/or unstable self‐image	Present	Present (only in the mania)
7. Marked impulsiveness	Present	Present (only in the mania)
8. Persistent feelings of emptiness	Present	Present (only in the depression)
9. Sudden anger, irritability, and/or unwarranted aggression	Present	Present
10. Irrational thoughts and beliefs	Present	Present (only in the mania)
11. Manic/hypomanic episodes	Present	Present (only in the mania)
12. Depressive/dysthymic episodes	Present	Present (only in the depression)
13. Low tolerance to frustration	Present	Present
14. Low tolerance to criticism	Present	Present (only in the mania)

### 4.4 BB‐co in PICI‐3

The topic is quite complex, as it intersects the combination of manic, bipolar, and borderline traits in its answer, as it can be inferred from the above structure that most of the commonalities between BPD and BpD are predominantly with manic tendency. Therefore, by superimposing the Manic‐Bipolar scale with the Bipolar‐Border scale, this question can be answered.

PICI‐TA‐3, the questionnaire that identifies dysfunctional personality traits only,^6^ presents the nosography of the two disorders separately, precisely because of their inherent characteristics, although they share several aspects in common, such as affective and emotional instability; however, the other differences are obvious: in the bipolar patient, there is a marked tendency toward emotional and affective instability that is more rigid and enriched by irrational beliefs taken to extremes, between depressive and manic episodes that represent the patient's two main modes (albeit with four different hypotheses that make the picture closer to one or the other); in the borderline patient, there is a greater and more pronounced tendency toward impulsivity and aggression, with emotional and affective fluctuations that are more abrupt and rapid, and depressive and manic episodes that are shorter and more circumscribed in time and space.

Therefore, it is more than correct to separate the two interpretative hypotheses, precisely because of their peculiar characteristics of the behavioral and emotional‐affective framework. By using the combined “Bipolar‐Border” (BB‐co) scale, which is the basis of the “Perrotta Border‐Bipolar Profile Diagnostic Questionnaire (PBBSD‐Q)”, it is possible to formulate the most appropriate diagnosis, as indicated in the interpretive rules for scoring the questionnaire (Attached 1).

## 5 DIAGNOSTIC PARALLELS BETWEEN BORDERLINE AND BIPOLAR PATIENTS IN PSYCHOPATHOLOGY AND NEUROBIOLOGICAL PROFILES

Neurobiological and genetic evidence confirms and reinforces the view that there are much deeper links between borderline disorder and BSD than we think and know today.^30^, ^31^, ^32^, ^33^, ^34^, ^35^, ^36^, ^37^, ^38^, ^39^, ^40^ In borderline disorder, the hyperactivated areas are the left amygdala and the white matter of the corpus callosum (which also have a smaller volume of gray matter), the insula and the posterior cingulate cortex, while the hypoactivated areas are the dorsolateral prefrontal cortex, the orbitofrontal cortex, the anterior cingulate cortex and the temporal‐occipital areas (which all have a smaller volume of gray matter), the nucleus accumbens and the hippocampus.^30^, ^34^ In BSD, there is hyperactivation of the hippocampus and hypoactivation and hypovolumetria of the bilateral locus coeruleus and white matter of the medial prefrontal cortex (with up to 40% reduction in glial cell number) and dorsolateral cortex (with up to 30% reduction in glial cell density at the laminin‐specific level), and subsequent impairment of glucose metabolism^30^, ^41^, ^42^, ^43^, ^44^, ^45^, ^46^; these impairments are related to dysfunctional levels of norepinephrine in the neocortical or subcortical limbic regions, (also responsible for mania), and if a functional alteration of serotonin (responsible for anxiety and depressive states) also occurs, this condition could explain with a neurobiological model why the mood swings typical of bipolar occur.^9^, ^13^, ^30^, ^31^, ^32^, ^33^, ^34^, ^35^, ^36^, ^37^, ^38^, ^39^, ^40^, ^41^, ^42^, ^43^, ^44^, ^45^, ^46^, ^47^, ^48^

## 6 PSYCHOMETRIC PROPOSAL: PBBSD‐Q

The new psychometric proposal takes its cue from the structure and operation of the PICI‐3,^6^ which sees the need to frame psychiatric symptomatology differently in specific personality disorders. Specifically, during experiments in clinical settings, the difficulty of nosographically framing the bipolar and borderline patient, especially in the presence of shared symptoms, was recurrent in the first and second versions; for this reason, the third version provided a technical adjustment to facilitate these diagnoses. Based on this assumption, the current psychometric proposal extends the scope by trying to coordinate the scoring rules in the presence of not only borderline and bipolar traits but also manic and depressive traits, identifying specific interpretive rules to define the exact nature of the psychopathological disorder, in a unified manner (Perrotta Border‐Bipolar Diagnostic Theory, PBBSD‐T), the prevalence of features per individual disorder, identifying 6 types for borderline disorder and 5 for BSD (Perrotta Border‐Bipolar Diagnostic Model, PBBSD‐M) and their symptom severity scales (Perrotta Border‐Bipolar Diagnostic Scale, PBBSD‐S) (Attached 1).

Based on these compatibility rules, the PBBSD‐Q was developed, structured into 36 items with dichotomous yes/no (Y/N) responses, with 9 progressive items per 5 categories (items 1–9 for manic traits, items 10–18 for bipolar traits, items 19–27 for depressive traits, 28–36 for emotive traits and items 37–45 for borderline traits) for 4 columns (A, B, C, D) corresponding to the 4 time reference periods (1‐2‐3‐4 months) from the day of administration. The therapist will manage the administration and the patient should answer the questions, with his/her support, choosing from 2 possible answers (Y for affirmative answer and N for negative answer) and referring to his/her personal experience of the last month of life (column A), of the month of life preceding that referred to column A (column B), of the month of life still preceding that referred to column B (column C), and finally of the month of life preceding that referred to column C (column D). An affirmative answer will be initialed when that behavior described in the item has a frequency of at least 7 out of 30 days. It is necessary, therefore, for each item to be answered 4 times to cover the last 4 months of life. Missing responses are not allowed (Attached 2).

## 7 DISCUSSION, CLINICAL APPLICATIONS AND PROSPECTS

The literature in recent decades has often questioned the need to clarify the relationship between borderline disorder and BSD, even going so far as to argue for their coexistence, as the former is a personality disorder while the latter is a mood disorder, and therefore they can be diagnosed together.^49^, ^50^

PICI‐3 completely overturns this point of view by accepting the perplexities in the clinical setting, on such an approach, and tries to clarify the importance of a more patient‐centered diagnosis, especially because of the clinical implications (and applications) that follow, starting from the psychotherapy to be set to the most appropriate drug therapy, which cannot and should no longer be based on the patient's narrative alone or on facts and circumstances that hardly ever represent the totality of the patient's personological complexity. Drug therapy for borderline and BSD must always be prescribed in constancy with psychotherapeutic courses to best benefit from cognitive remodeling effects.

Here then, with this proposal, an attempt has been made to offer a psychometric tool that can accommodate all these instances and allow the therapist a better, more systematic and accurate approach to the problem. The prospects of this proposal have a spin‐off in the clinical setting, in that it can facilitate a better diagnosis and thus a better social‐health approach to the diagnostic problem of these patients.

On the other hand, borderline disorder and BSD, per PICI‐3, are both personality disorders, and therefore the same prognosis, in terms of cognitive restructuring through clinical intervention, depends on several factors: age (the greater the age the lower the chance of successful clinical intervention), sexual gender (depending on the disorder there is a lower or higher prevalence), attachment style, and traumatic events in childhood and during life development that were not functionally reprocessed, the severity of symptomatology (in terms of symptomatological expression in daily life and how it negatively impacts one's own and other's lives), socio‐personal and family context, life circumstances that have hyperactivating power, the centrality of the diagnosis to the profile, prescribed clinical therapy, and awareness of one's status and need for change. A more detailed and structured nosography between borderline disorders and BSD facilitates the clinical framing of the patient, promoting a lower risk of diagnostic errors, both in terms of diagnosis and treatment. An experiment is already underway with a representative sample to evaluate the suitability of the instrument, and thus its validity in statistical terms.

In the Table 3, the clinical messages, as a result of the literature search, were presented (Table 3).

**Table 3:** Clinical messages.

Keyword	Clinical message
*Etiology*	Borderline disorder and bipolar spectrum disorder are strongly interconnected, as both are neurobiological.
*Diagnosis*	The diagnosis of borderline disorder and bipolar spectrum disorder, in those patients who both present with psychopathological features, must also be confronted with any manic and depressive traits, which may not be fully included in bipolar spectrum disorder as they have less impact on the patient's personality profile. Having a nosographic that takes these aspects into account is of primary importance.
*Therapy*	Drug therapy for borderline and bipolar spectrum disorder must always be prescribed in constancy with psychotherapeutic course, to best benefit from the cognitive remodeling effects. Having a nosography that considers the relationships between these disorders is of paramount importance.
*Prognosis*	Borderline disorder and bipolar spectrum disorder, per PICI‐3, are both personality disorders, and therefore the prognosis, in terms of cognitive restructuring through clinical intervention, depends on several factors: age, sexual gender, attachment style, traumatic events in childhood and during life development that have not been functionally reprocessed, severity of symptomatology, socio‐personal and family context, life circumstances that have hyperactivating power, centrality of diagnosis to profile, prescribed clinical therapy, awareness of one's state and need for change.
*Psychological implications*	A more detailed and structured nosography between borderline disorders and bipolar spectrum disorder facilitates the clinical framing of the patient, promoting a lower risk of diagnostic errors, both in terms of diagnosis and treatment.

## 8 CONCLUSIONS

The present research attempts to provide the reader and the health professional with a comprehensive overview of borderline and BSD, according to the PICI model, highlighting some aspects that could redefine their clinical framing, thus facilitating a better diagnostic approach to the problem and offering an innovative key that could eliminate the problem of diagnostic error at its root, through the proposal of a questionnaire PBBSD‐Q, which could refine the diagnosis in the hypotheses of borderline disorder and BSD once the validation process is successful. The intent of framing the Border‐Bipolar universe in a more structured and functional way, offering both a theory and a model that considers the relevant literature and an already organized questionnaire for diagnosis, guarantees mental health professionals a psychometric tool, with clear, comprehensive interpretive rules consistent with the descriptive framework of the patient.

## AUTHOR CONTRIBUTIONS

Giulio Perrotta conceived the theory, the model, and the related questionnaire, drafting the manuscript. He holds the exclusive rights of paternity and exploitation on the conception of the theory, the model, and the questionnaire. Simona Grilli, Stefano Eleuteri, and Irene Petruccelli contributed to the revision of the manuscript.

## CONFLICT OF INTEREST STATEMENT

The authors declare no conflicts of interest.

## ETHICS STATEMENT

Not applicable.

## Supporting information

Supporting information.

## Data Availability

All data supporting the findings of this study are available within the review and its supplementary materials.
