# Supplementary material for: Diagnostic parallels between borderline and bipolar patients in psychopathology: Similarities, differences, comorbidities, neural correlates, and a new proposal for the Perrotta Border‐Bipolar Profile Diagnostic Questionnaire
Source: Ibrain. 2025 Sep 2;11(3):306–18. doi: 10.1002/ibra.70001 (PMC12465228; doi:10.1002/ibra.70001)
Supplement: Supplementary file 1 — Supporting information. [file IBRA-11-306-s001.docx]

**Attached 1**:

*Perrotta Border-Bipolar Profile Diagnostic Questionnaire (PBBSD-Q). Scoring rules*

| The questionnaire is divided into 5 sections (sub-1, sub-2, sub-3, sub-4, sub-5) and each section consists of 9 items with Y/N (true/false) responses for 4 columns (A-B-C-D), corresponding to 4 different periods. Answer the items, with the support of the trained therapist, choosing from the 2 possible answers (Y for yes answer and N for no answer) and referring to one's personal experience of the last month of life (column A), of the month of life preceding the one referring to column A (column B), of the month of life still preceding the one referring to column B (column C), and finally of the month of life preceding the one referring to column C (column D). Thus, each item must be answered 4 times to cover the last 4 months of life. An affirmative answer will be initialed when that behavior described in the item has a frequency of at least 7 out of 30 days. It is necessary, therefore, for each item to be answered 4 times to cover the last 4 months of life. Missing responses are not allowed. Scoring will be done by the therapist considering the following rules:   1. Section SUB-1 (items 1-9) is devoted to the 0-9 score of the manic scale. 2. Section SUB-2 (items 10-18) is devoted to the 0-9 score of the bipolar scale. 3. Section SUB-3 (items 19-27) is devoted to the 0-9 score of the emotive scale 4. Section SUB-4 (items 28-36) is devoted to the 0-9 score of the depressive scale. 5. Section SUB-5 (items 37-45) is devoted to the 0-9 score of the borderline scale. 6. Adding up the positive scores for each column and each section, the therapist will use the following grid (combined "Bipolar-Border" (BB-co) scale, for the diagnosis of the Bipolar-Border condition, using the “Perrotta Border-Bipolar Profile Diagnostic Questionnaire”, PBBSD-Q):  \|  \| **Manic**  **(scale no. 6)** \| **Bipolar**  **(scale no. 7)** \| **Emotive**  **(scale no. 8)** \| **Depressive**  **(scale no. 10)** \| **Borderline**  **(scale no. 11)** \| **Final Diagnosis** \| \| --- \| --- \| --- \| --- \| --- \| --- \| --- \| \| **N_ dysfunctional traits**  **(PICI-TA-3)** \| < 9/9 \| 9/9 \| ≤ 9/9 \| < 9/9 \| \| = Bipolar \| \| --- \| \| < Bipolar \| \| Borderline  Bipolar \| \| < 8/9 \| ≥ 8/9 \| ≤ 8/9 \| < 8/9 \| \| ≥ Bipolar \| \| --- \| \| < Bipolar \| \| Borderline  Bipolar \| \| < 7/9 \| ≥ 7/9 \| ≤ 7/9 \| < 7/9 \| \| ≥ Bipolar \| \| --- \| \| < Bipolar \| \| Borderline  Bipolar \| \| < 6/9 \| ≥ 6/9 \| ≤ 6/9 \| < 6/9 \| \| ≥ Bipolar \| \| --- \| \| < Bipolar \| \| Borderline  Bipolar \| \| 9/9 \| 9/9 \| 9/9 \| < 9/9 \| \| = Bipolar \| \| --- \| \| < Bipolar \| \| Borderline  Bipolar \| \| 8/9 \| 8/9 \| 8/9 \| < 8/9 \| \| ≥ Bipolar \| \| --- \| \| < Bipolar \| \| Borderline  Bipolar \| \| 7/9 \| 7/9 \| 7/9 \| < 7/9 \| \| ≥ Bipolar \| \| --- \| \| < Bipolar \| \| Borderline  Bipolar \| \| 6/9 \| 6/9 \| 6/9 \| < 6/9 \| \| ≥ Bipolar \| \| --- \| \| < Bipolar \| \| Borderline  Bipolar \| \| = Bipolar \| = Manic \| ≤ Bipolar \| < Bipolar \| \| ≥ Bipolar \| \| --- \| \| < Bipolar \| \| Borderline  Bipolar, markedly manic \| \| < Bipolar \| = Depressive \| ≤ Bipolar \| = Bipolar \| \| ≥ Bipolar \| \| --- \| \| < Bipolar \| \| Borderline  Bipolar, markedly depressive \| \| > Bipolar \| < Manic \| ≤ / ≥ Manic \| < Manic \| \| ≥ Manic \| \| --- \| \| < Manic \| \| Borderline  Manic \| \| < Depressive \| < Depressive \| ≤ / ≥ Depressive \| > Bipolar \| \| ≥ Depressive \| \| --- \| \| < Depressive \| \| Borderline  Depressive \| \| = Depressive \| < Depressive \| ≤ / ≥ Depressive \| = Manic \| \| ≥ Depressive \| \| --- \| \| < Depressive \| \| Borderline  Bipolar (mixed or atopic) \| \| ≤ 5/9 \| 5/9 \| ≤ 5/9 \| ≤ 5/9 \| \| = Bipolar \| \| --- \| \| < Bipolar \| \| Borderline  Manic-depressive elevation (or white bipolarity) \| \| 5/9 \| 5/9 \| ≤ 5/9 \| < 5/9 \| \| = Bipolar \| \| --- \| \| < Bipolar \| \| Borderline  White bipolarity, markedly manic \| \| < 5/9 \| 5/9 \| ≤ 5/9 \| 5/9 \| \| = Bipolar \| \| --- \| \| < Bipolar \| \| Borderline  White bipolarity, markedly depressive \| \| < 5/9 \| < 5/9 \| ≥ 5/9 \| < 5/9 \| \| ≥ Emotive \| \| --- \| \| < Emotive \| \| Borderline  Emotive \| \| = Emotive \| < 5/9 \| ≥ 5/9 \| < 5/9 \| \| ≥ Emotive \| \| --- \| \| < Emotive \| \| Borderline  Emotive, markedly manic \| \| < 5/9 \| < 5/9 \| ≥ 5/9 \| = Emotive \| \| ≥ Emotive \| \| --- \| \| < Emotive \| \| Borderline  Emotive, markedly depressive \| \| < 4/9 \| 4/9 \| ≤ 4/9 \| < 4/9 \| \| = Bipolar \| \| --- \| \| < Bipolar \| \| Bipolar predisposition (humoral fragility) \| \| 4/9 \| 4/9 \| ≤ 4/9 \| < 4/9 \| \| = Bipolar \| \| --- \| \| < Bipolar \| \| \| 4/9 \| 4/9 \| ≤ 4/9 \| < 4/9 \| \| = Bipolar \| \| --- \| \| < Bipolar \| \| Humoral fragility,  markedly manic \| \| < 4/9 \| 4/9 \| ≤ 4/9 \| 4/9 \| \| = Bipolar \| \| --- \| \| < Bipolar \| \| Humoral fragility,  markedly depressive \| \| < 3/9 \| 3/9 \| ≤ 3/9 \| < 3/9 \| \| = Bipolar \| \| --- \| \| < Bipolar \| \| Bipolar inclination (humoral tendency) \| \| 3/9 \| 3/9 \| ≤ 3/9 \| < 3/9 \| \| = Bipolar \| \| --- \| \| < Bipolar \| \| \| 3/9 \| 3/9 \| ≤ 3/9 \| < 3/9 \| \| = Bipolar \| \| --- \| \| < Bipolar \| \| Humoral tendency,  markedly manic \| \| < 3/9 \| 3/9 \| ≤ 3/9 \| 3/9 \| \| = Bipolar \| \| --- \| \| < Bipolar \| \| Humoral tendency,  markedly depressive \| \| < 3/9 \| < 3/9 \| < 3/9 \| < 3/9 \| < 3/9 \| No pathological significance \|  1. In filling in the total score, Section A (blue box) refers to itself and indicates the diagnosis of the last trimester, Section B+C+D (red box) refers to the unit diagnosis of the remaining (past) period, and Section E (light blue box) refers to the total unit diagnosis (A+B+C+D). For the total diagnosis of Section A, reference should be made to the table above. For the total diagnosis (B+C+D) and overall total (A+B+C+D), reference should be made to the following table:  \| **N** \| **Hypothesis** \| **Diagnosis** \| \| --- \| --- \| --- \| \| 1 \| At least 1 column presents "Bipolar" but not "Borderline" \| Bipolar \| \| 2 \| At least 1 column presents "Bipolar" and "Manic" but not "Borderline" and not "Depressive" \| Bipolar markedly manic \| \| 3 \| At least 1 column presents "Bipolar" and "Depressive", but not "Borderline" and not "Manic" \| Bipolar markedly depressive \| \| 4 \| At least 1 column presents "Bipolar", "Manic", and "Depressive", but not "Borderline" \| Bipolar \| \| 5 \| If at least 1 column has "Borderline" \| Borderline \| \| 6 \| If "Emotional" is present, this is automatically absorbed by "Bipolar" or "Borderline", in their absence the words "Markedly Manic" or "Markedly Depressive Emotional" remain \| \| \| 7 \| Equal score or lower: Borderline absorbs all other categories; Bipolar absorbs all but Borderline; categories with scores < 5/9 are automatically absorbed by Borderline and Bipolar \| \| \| 8 \| If the score is <3/9 the words "Absence of pathological significance" will be assigned \| \|   Finally, to define the specific type of "Borderline" and "Bipolar", reference should be made to the following table. Up to 2 different types can coexist (e.g., unstable-impulsive and deflected):   \| **BORDERLINE** \| \| \| \| --- \| --- \| --- \| \| TYPE 1 \| UNSTABLE-IMPULSIVE \| Positivity to at least 5/11 items:  1-4-8-12-14-21-22-29-32-38-40  The patient manifests a prevalence of impulsive behaviors that outline an unstable and insecure personality, continually seeking emotional and/or practical caretaking and validation (e.g., financial maintenance or continual economic demands or practical advantages). They may be prone to alcohol and drug use. This profile has more relatedness and similarities with manic traits. \| \| TYPE 2 \| AGGRESSIVE-EXPLOSIVE \| Positivity to at least 6/12 items:  3-5-10-16-17-23-24-37-39-40-43-44  The patient manifests a prevalence of aggressive, violent and explosive behaviors in circumstances that are insufficient to justify the reaction, outlining a constellation of personality traits of the insecure, arrogant, angry, threatening and conceited type, constantly seeking emotional validation through the power and in severe cases even resorting to violence (verbal and/or physical). They may be prone to alcohol and drug use. This profile has more relatedness and similarities with the manic, narcissistic and antisocial-psychopathic traits. \| \| TYPE 3 \| EMOTIONAL-HUMORAL \| Positivity to at least 6/13 items:  6-7-9-11-13-15-20-25-26-27-39-41-44  The patient manifests a prevalence of emotional behaviors, outlining an excessively moody personality prone to excesses, constantly seeking confirmation of value and ability. They may be prone to alcohol and drug use. This profile has more similarities and resemblances to neurotic, emotional and bipolar traits. \| \| TYPE 4 \| DEFLECTED \| Positivity to at least 4/7 items:  28-30-31-33-35-36-42  The patient manifests a prevalence of passive-aggressive behaviors with marked depressive tendencies, constant requests for help and suicidal tendencies, outlining an unstable and fragile personality, constantly seeking attention. They may be prone to alcohol and drug use. This profile has more relatedness and similarities with depressive and bipolar traits. \| \| TYPE 5 \| FRAGMENTED \| Positivity to at least 5/10 items:  2-17-18-19-31-32-34-36-43-45  The patient manifests a prevalence of fragmented behavior, with detachment from reality and marked psychotic symptomatology (dissociation, delusions, paranoia, obsessions and mania), outlining an insecure and fragile personality, constantly seeking protection. They may be prone to alcohol and drug use. This profile has more similarities and similarities with psychotic traits. \| \| TYPE 6 \| MIXED \| Positivity to at least 22/45 items, with no fulfilled any of the requirements for the other types  The patient manifests a constellation of heterogeneous traits of 2 or more of the types listed above, which do not meet individual interpretive rules but collectively represent a dysfunctional personality. They may be prone to alcohol and drug use. This profile has more relatedness and similarities with bipolar, narcissistic, histrionic, and psychopathic traits. \| \| **BIPOLAR** \| \| \| \| TYPE 1 \| MANIACAL-DEPRESSIVE (or PURE) \| The patient alternates cyclically:   1. a period of abnormally and persistently elevated, expansive or irritable mood lasting at least 7 days (manic episode) and up to 3-4 months, with at least 3 symptoms among: (a1) excessive self-esteem or delusions of grandeur; (a2) Reduced need for sleep; (a3) greater talkativeness than usual, or continuous urge to talk; (a4) flight of ideas; (a5) distractibility (attention too easily diverted by unimportant or irrelevant external stimuli); (a6) mental and motor agitation; (a7) excessive involvement in activities with sometimes harmful consequences such as shopping, unseemly sexuality, or investment. 2. a period of abnormally and persistently low mood, lasting for at least 7 days (depressive episode) and up to 3-4 months, with at least 6 symptoms among: b1) sadness, hopelessness and feeling of emptiness; b2) weeping; b3) irritability (especially in younger people); b4) loss of interest in all those activities, which usually represent a fun or appreciated pastime; b5) weight changes; b6) disturbance of night sleep (e.g: insomnia or excessive sleepiness); b7) lethargy; b8) sense of worthlessness or guilt; b9) restlessness; b10) sense of fatigue and lack of energy; b11) slowness in doing things; b12) feeling useless and having senses of strokes for no reason; b13) concentration problems; b14) suicidal thoughts. \| \| TYPE 2 \| MANIC-DYSTHYMIC \| The patient alternates cyclically:   1. a period of abnormally and persistently elevated, expansive or irritable mood lasting at least 7 days (manic episode) and up to 3-4 months, with at least 3 symptoms among: (a1) excessive self-esteem or delusions of grandeur; (a2) Reduced need for sleep; (a3) greater talkativeness than usual, or continuous urge to talk; (a4) flight of ideas; (a5) distractibility (attention too easily diverted by unimportant or irrelevant external stimuli); (a6) mental and motor agitation; (a7) excessive involvement in activities with sometimes harmful consequences such as shopping, unseemly sexuality, or investment. 2. a period of abnormally and persistently low mood, lasting for about 3-7 days (depressive episode) and up to 1-2 months, with at least 6 symptoms among: b1) sadness, hopelessness and feeling of emptiness; b2) weeping; b3) irritability (especially in younger people); b4) loss of interest in all those activities, which usually represent a fun or appreciated pastime; b5) weight changes; b6) disturbance of night sleep (e.g: insomnia or excessive sleepiness); b7) lethargy; b8) sense of worthlessness or guilt; b9) restlessness; b10) sense of fatigue and lack of energy; b11) slowness in doing things; b12) feeling useless and having senses of strokes for no reason; b13) concentration problems; b14) suicidal thoughts. \| \| TYPE 3 \| HYPOMANIC-DEPRESSIVE \| The patient alternates cyclically:   1. a period of abnormally and persistently elevated, expansive or irritable mood, lasting about 3-7 days (manic episode) and up to 1-2 months, with at least 3 symptoms among: (a1) excessive self-esteem or delusions of grandeur; (a2) Reduced need for sleep; (a3) increased talkativeness than usual, or continuous urge to talk; (a4) flight of ideas; (a5) distractibility (attention too easily diverted by unimportant or irrelevant external stimuli); (a6) mental and motor agitation; (a7) excessive involvement in activities with sometimes harmful consequences such as shopping, unseemly sexuality, or investment. 2. a period of abnormally and persistently low mood, lasting for at least 7 days (depressive episode) and up to 3-4 months, with at least 6 symptoms among: b1) sadness, hopelessness and feeling of emptiness; b2) weeping; b3) irritability (especially in younger people); b4) loss of interest in all those activities, which usually represent a fun or appreciated pastime; b5) weight changes; b6) disturbance of night sleep (e.g: insomnia or excessive sleepiness); b7) lethargy; b8) sense of worthlessness or guilt; b9) restlessness; b10) sense of fatigue and lack of energy; b11) slowness in doing things; b12) feeling useless and having senses of strokes for no reason; b13) concentration problems; b14) suicidal thoughts. \| \| TYPE 4 \| HYPANIACAL-DYSTIMIAN (or CYCLOTHYMIAN) \| The patient alternates cyclically:   1. a period of abnormally and persistently elevated, expansive or irritable mood, lasting about 3-7 days (manic episode) and up to 1-2 months, with at least 3 symptoms among: (a1) excessive self-esteem or delusions of grandeur; (a2) Reduced need for sleep; (a3) increased talkativeness than usual, or continuous urge to talk; (a4) flight of ideas; (a5) distractibility (attention too easily diverted by unimportant or irrelevant external stimuli); (a6) mental and motor agitation; (a7) excessive involvement in activities with sometimes harmful consequences such as shopping, unseemly sexuality, or investment. 2. a period of abnormally and persistently low mood, lasting for about 3-7 days (depressive episode) and up to 1-2 months, with at least 6 symptoms among: b1) sadness, hopelessness and feeling of emptiness; b2) weeping; b3) irritability (especially in younger people); b4) loss of interest in all those activities, which usually represent a fun or appreciated pastime; b5) weight changes; b6) disturbance of night sleep (e.g: insomnia or excessive sleepiness); b7) lethargy; b8) sense of worthlessness or guilt; b9) restlessness; b10) sense of fatigue and lack of energy; b11) slowness in doing things; b12) feeling useless and having senses of strokes for no reason; b13) concentration problems; b14) suicidal thoughts. \| \| TYPE 5 \| MIXED \| The patient alternates cyclically between both manic and hypomanic periods with depressive and dysthymic periods, with a frequency that does not fit into any of the previous types. \|   The measure of the severity of the morbid condition is determined by the following rule: for the identified type, positivity up to 25% of the reference items identifies the judgment of "mild", positivity between 26% and 50% of the reference items identifies the judgment of "moderate", positivity between 51% and 75% of the reference items identifies the judgment of "severe", and positivity between 76% and 100% of the reference items identifies the judgment of "severe". |
| --- | --- | --- | --- | --- | --- | --- | --- | --- | --- | --- | --- | --- | --- | --- | --- | --- | --- | --- | --- | --- | --- | --- | --- | --- | --- | --- | --- | --- | --- | --- | --- | --- | --- | --- | --- | --- | --- | --- | --- | --- | --- | --- | --- | --- | --- | --- | --- | --- | --- | --- | --- | --- | --- | --- | --- | --- | --- | --- | --- | --- | --- | --- | --- | --- | --- | --- | --- | --- | --- | --- | --- | --- | --- | --- | --- | --- | --- | --- | --- | --- | --- | --- | --- | --- | --- | --- | --- | --- | --- | --- | --- | --- | --- | --- | --- | --- | --- | --- | --- | --- | --- | --- | --- | --- | --- | --- | --- | --- | --- | --- | --- | --- | --- | --- | --- | --- | --- | --- | --- | --- | --- | --- | --- | --- | --- | --- | --- | --- | --- | --- | --- | --- | --- | --- | --- | --- | --- | --- | --- | --- | --- | --- | --- | --- | --- | --- | --- | --- | --- | --- | --- | --- | --- | --- | --- | --- | --- | --- | --- | --- | --- | --- | --- | --- | --- | --- | --- | --- | --- | --- | --- | --- | --- | --- | --- | --- | --- | --- | --- | --- | --- | --- | --- | --- | --- | --- | --- | --- | --- | --- | --- | --- | --- | --- | --- | --- | --- | --- | --- | --- | --- | --- | --- | --- | --- | --- | --- | --- | --- | --- | --- | --- | --- | --- | --- | --- | --- | --- | --- | --- | --- | --- | --- | --- | --- | --- | --- | --- | --- | --- | --- | --- | --- | --- | --- | --- | --- | --- | --- | --- | --- | --- | --- | --- | --- | --- | --- | --- | --- | --- | --- | --- | --- | --- | --- | --- | --- | --- | --- | --- | --- | --- | --- | --- | --- | --- | --- | --- | --- | --- | --- | --- | --- | --- | --- | --- | --- | --- | --- | --- | --- | --- | --- | --- | --- | --- | --- | --- | --- | --- | --- | --- | --- | --- |

**Attached 2:**

*Perrotta Border-Bipolar Profile Diagnostic Questionnaire (PBBSD-Q)*

| *Perrotta Border-Bipolar Profile Diagnostic Questionnaire*  **PBBSD-Q** | | | | | | | | | | | |
| --- | --- | --- | --- | --- | --- | --- | --- | --- | --- | --- | --- |
| **N** | **ITEM** | | | | **A** | **B** | | **C** | | **D** | |
| 1 | Over the day, do you repeatedly feel tense and/or agitated, with no real reason capable of justifying these emotional states? | | | | Y N | Y N | | Y N | | Y N | |
| 2 | Have you ever been convinced of something wrong but continued to believe it to be true? | | | | Y N | Y N | | Y N | | Y N | |
| 3 | Do you notice or are you noticed at certain times that you are particularly distractible or have deficits relative to attention and short-term memory? | | | | Y N | Y N | | Y N | | Y N | |
| 4 | Do you tend to be unthrifty and/or otherwise engage in potentially harmful and/or dangerous activities (compulsive shopping, unseemly sexuality, hasty investments, substance taking)? | | | | Y N | Y N | | Y N | | Y N | |
| 5 | Do you feel that your ideas to travel fast, overlap, are uncontrollable, and/or leave one or more started tasks unfinished? | | | | Y N | Y N | | Y N | | Y N | |
| 6 | Do you feel several times a day need to lock yourself in your home or office to work, even well past working hours and/or canceling appointments? | | | | Y N | Y N | | Y N | | Y N | |
| 7 | Do you believe that your ideas are brilliant or otherwise important or do you otherwise perceive that your self-esteem is out of control, or do you have an optimism that is not compatible with reality? | | | | Y N | Y N | | Y N | | Y N | |
| 8 | Do you feel hyperactive at certain times of the day or do those around you point it out? | | | | Y N | Y N | | Y N | | Y N | |
| 9 | Do you feel at certain times that you are logorrheic or otherwise overly talkative? | | | | Y N | Y N | | Y N | | Y N | |
| **TOTAL SUB-1** | | | | | ___ / 9 | ___ / 9 | | ___ / 9 | | ___ / 9 | |
| 10 | Do you get irritated easily, without a serious enough reason to justify this emotional state? | | | | Y N | Y N | | Y N | | Y N | |
| 11 | Do you feel that daily worries or tensions are crushing you? | | | | Y N | Y N | | Y N | | Y N | |
| 12 | Do you feel that your mood is not always stable? | | | | Y N | Y N | | Y N | | Y N | |
| 13 | Do you feel emotionally unstable? | | | | Y N | Y N | | Y N | | Y N | |
| 14 | Are your social relationships affected by your mood? | | | | Y N | Y N | | Y N | | Y N | |
| 15 | Have you ever felt, either sad or elated, for a medium-too long cyclical period? | | | | Y N | Y N | | Y N | | Y N | |
| 16 | Have you ever tried to actively obtain something against the other person's will? | | | | Y N | Y N | | Y N | | Y N | |
| 17 | Do you get hurt by criticism, even if it is deserved? | | | | Y N | Y N | | Y N | | Y N | |
| 18 | Do you tend to have more frequent unpleasant feelings and/or a tendentially pessimistic mode? | | | | Y N | Y N | | Y N | | Y N | |
| **TOTAL SUB-2** | | | | | ___ / 9 | ___ / 9 | | ___ / 9 | | ___ / 9 | |
| 19 | Do you sense or are you reported to tend to be overly rigid in your thinking and/or to stand firm in your positions even when you might be wrong? | | | | Y N | Y N | | Y N | | Y N | |
| 20 | Do you sense or are told that you tend to have poor frustration management, which causes you to be easily irritable? | | | | Y N | Y N | | Y N | | Y N | |
| 21 | Do you tend to violate social norms and norms of civil coexistence or otherwise fail to perform the diligence that would be expected in the social setting? | | | | Y N | Y N | | Y N | | Y N | |
| 22 | Do you tend to violate legal norms or otherwise fail to perform the diligence that would be expected in the relevant context? | | | | Y N | Y N | | Y N | | Y N | |
| 23 | Have you received reprimands, reprimands or punishments because of your excessive behaviors? | | | | Y N | Y N | | Y N | | Y N | |
| 24 | Have you manifested explosive, uncontrolled and/or unjustified verbal anger compared to the circumstances? | | | | Y N | Y N | | Y N | | Y N | |
| 25 | Do you sense or are told that you tend to have complicated control/management of your internal emotional plane or otherwise feel that you are in disharmony with what you feel? | | | | Y N | Y N | | Y N | | Y N | |
| 26 | Do you sense or are you told that you tend to make certain choices instinctively (i.e., reasoned impulsiveness), risking exposure to criticism and judgment for your behavior? | | | | Y N | Y N | | Y N | | Y N | |
| 27 | Do you perceive or are you reported to perceive that you tend not infrequently to have childish and/or wayward attitudes, in any case not in keeping with the circumstances, your age, your social position, and/or in general your environment of reference? | | | | Y N | Y N | | Y N | | Y N | |
| **TOTAL SUB-3** | | | | | ___ / 9 | ___ / 9 | | ___ / 9 | | ___ / 9 | |
| 28 | Do you get tired mentally and/or physically easily and/or feel that you cannot finish all the daily scheduled activities? | | | | Y N | Y N | | Y N | | Y N | |
| 29 | Have you experienced weight loss and/or sleep-wake rhythm alterations without a specific cause? | | | | Y N | Y N | | Y N | | Y N | |
| 30 | Would you describe your mood as tending to or always sad? | | | | Y N | Y N | | Y N | | Y N | |
| 31 | Do you perceive, over the day, one or more episodes of a marked decrease in pleasure in carrying out interests and activities? | | | | Y N | Y N | | Y N | | Y N | |
| 32 | Do you perceive, over the day, one or more episodes of marked boredom and/or disinterest, although you have interesting activities you can do? | | | | Y N | Y N | | Y N | | Y N | |
| 33 | Have you had or have bouts of crying, sadness, and/or emptiness not otherwise justified? | | | | Y N | Y N | | Y N | | Y N | |
| 34 | Have you experienced psychomotor slowdown because of your mood? | | | | Y N | Y N | | Y N | | Y N | |
| 35 | Do you frequently experience feelings of inappropriateness, self-evaluation, and/or marked guilt in the absence of a justifiable cause? | | | | Y N | Y N | | Y N | | Y N | |
| 36 | Do you frequently experience negative or melancholic and/or death-related thoughts that are not provoked by actual events? | | | | Y N | Y N | | Y N | | Y N | |
| **TOTAL SUB-4** | | | | | ___ / 9 | ___ / 9 | | ___ / 9 | | ___ / 9 | |
| 37 | Have you ever tried to get passive-aggressive attitudes against the other person's will? | | | | Y N | Y N | | Y N | | Y N | |
| 38 | Do you react to life events impulsively? | | | | Y N | Y N | | Y N | | Y N | |
| 39 | Do you feel that people, after getting to know you, tend to push you away and/or put up boundaries? | | | | Y N | Y N | | Y N | | Y N | |
| 40 | Do you do what you can, putting your all into it, to keep people from turning away from you and/or abandoning you? | | | | Y N | Y N | | Y N | | Y N | |
| 41 | Do you feel that your true being cannot come out externally and/or is better off not coming out and/or will not be understood if it comes out externally? | | | | Y N | Y N | | Y N | | Y N | |
| 42 | Do you perceive a sense of emptiness and/or boredom in you despite your daily activities and your family, friends, and work circle? | | | | Y N | Y N | | Y N | | Y N | |
| 43 | Have you ever been angry or aggressive unjustifiably and/or disproportionately to the offense or danger? | | | | Y N | Y N | | Y N | | Y N | |
| 44 | Have you ever had sudden anger without a definite trigger? | | | | Y N | Y N | | Y N | | Y N | |
| 45 | Have you ever felt, either sad or elated, for a short period even daily? | | | | Y N | Y N | | Y N | | Y N | |
| **TOTAL SUB-5** | | | | | ____ / 9 | ____ / 9 | | ____ / 9 | | ____ / 9 | |
| **TOTAL PBBSD-Q** | | M: _____ / 9    B: ______ / 9    E: _____ / 9    D: _____ / 9    BSD: _____ / 9 | | M: _____ / 9    B: ______ / 9    E: _____ / 9    D: _____ / 9    BSD: _____ / 9 | | | M: _____ / 9    B: ______ / 9    E: _____ / 9    D: _____ / 9    BSD: _____ / 9 | | M: _____ / 9    B: ______ / 9    E: _____ / 9    D: _____ / 9    BSD: _____ / 9 | | |
|  |  | _____(p= )_  (**A**) | | _____(p= )_  (**B**) | | | _____(p= )_  (**C**) | | _____(p= )_  (**D**) | | |
| _______________________________________  (**E**) | | | | | | | |  |  |  |  |
